# mRNA expression of toll-like receptors 3, 7, 8, and 9 in the nasopharyngeal epithelial cells of coronavirus disease 2019 patients

**DOI:** 10.1186/s12879-022-07437-9

**Published:** 2022-05-10

**Authors:** Zahra Bagheri-Hosseinabadi, Ebrahim Rezazadeh Zarandi, Mohammad Mirabzadeh, Ali Amiri, Mitra Abbasifard

**Affiliations:** 1 Pistachio Safety Research Center, Rafsanjan University of Medical Sciences, Rafsanjan, Iran; 2 Department of Clinical Biochemistry, School of Medicine, Rafsanjan University of Medical Sciences, Rafsanjan, Iran; 3 Immunology of Infectious Diseases Research Center, Research Institute of Basic Medical Sciences, Rafsanjan University of Medical Sciences, Rafsanjan, Iran; 4 Student Research Committee, Rafsanjan University of Medical Sciences, Rafsanjan, Iran; 5 Department of Orthodontics, College of Stomatology, The First Affiliated Stomatological Hospital, Xi’an Jiaotong University, Xi’an, 710004 People’s Republic of China; 6 Department of Internal Medicine, Ali-Ibn Abi-Talib Hospital, School of Medicine, Rafsanjan University of Medical Sciences, Rafsanjan, Iran; 7 Department of Microbiology, School of Medicine, Rafsanjan University of Medical Sciences, Rafsanjan, Iran

**Keywords:** Severe acute respiratory syndrome coronavirus 2, Coronavirus disease 2019, Inflammation, Toll-like receptor, Epithelial cell

## Abstract

**Background:**

The etiopathogenesis of coronavirus disease 2019 (COVID-19) stem partially from the abnormal activation of the innate and adaptive immune systems. Here in the current investigation, the mRNA expression levels of toll-like receptors (TLRs) were evaluated in the nasopharyngeal epithelial cells from COVID-19 patients.

**Methods:**

Epithelial cells were obtained using nasopharyngeal swab samples from 90 COVID-19 patients and 50 controls. COVID-19 cases were classified into those without symptoms, with symptoms but not hospitalized, and with symptoms and hospitalized. To determine the mRNA expression levels of TLRs, first RNA was extracted and cDNA was synthesized, and finally Real-time PCR was exerted.

**Results:**

It was seen that the transcript levels of TLR3, TLR7, TLR8, and TLR9 were overexpressed in the COVID-19 patients with clinical symptoms needing hospitalization as well as in those with clinical symptoms without needing for hospitalization compared to controls. Upregulation of TLRs was associated with clinical presentations of the patients.

**Conclusions:**

Modulation of TLR3, TLR7, TLR8, TLR9 in the epithelial cells of COVID-19 cases may estimate the disease severity and requirement for hospitalization.

## Introduction

As a pandemic occurred in March 2020, coronavirus disease 2019 (COVID-19) caused by severe acute respiratory syndrome coronavirus 2 (SARS-CoV-2) is currently a global health and hygiene issue worldwide [1]. The precise mechanism of pathogenesis by SARS‐CoV‐2 and the function immune system against virus remains undivulged. SARS-CoV-2 spike protein via its receptor-binding domain (RBD) binds to its receptor on the human tissues, namely human angiotensin-converting enzyme 2 (hACE2) and is proteolytically activated by human proteases [2]. Although several subjects with SARS-CoV2 infection show a moderate form COVID-19 with less serious clinical presentations, about 10–15% of subjects develop a severe illness requiring hospitalization and supportive cares, and 5% of patients might require admission into intensive care units (ICU) [3, 4]. During the severe forms of COVID-19 disease, cytokine storm may result in acute respiratory distress syndrome (ARDS), sepsis, septic shock, multiorgan failure, and even death in the severe forms of the infection [5–8].

Toll‐like receptors (TLRs) play important roles in several processes of innate immune system, such as stimulation of innate immune system, indirect triggering of the adaptive immune system, and modulation of cytokine expression mainly through identification of pathogen‐associated molecular patterns (PAMPs) [9, 10]. TLR3 is involved in the identification of double‐strand RNA (dsRNA), TLR4 recognizes bacterial lipopolysaccharide (LPS), TLR7/8 identifies single‐strand RNA (ssRNA), and TLR9 identifies bacterial unmethylated CpG DNA [11]. Signaling transduction pathway of TLRs and are involved in stimulation of the Interferon regulatory factor (IRF) and nuclear factor (NF)‐κB, resulting in the generation of type 1 interferon (IFN) as well as pro‐inflammatory cytokines like interleukin (IL)‐1, IL‐6, IL-12, and tumor necrosis factor (TNF)‐α [12]. In addition, TLRs play indirect roles in the activation of the adaptive immune system through stimulating the expression of costimulatory molecules involved in activation of the B and T cells [13].

It has been reported that SARS-CoV-2 is able to stimulate the pro-inflammatory genes as well as interferon/cytokine signaling molecules in the lung epithelial cells, particularly club and ciliated cells, from COVID-19 patients [14]. Several viruses are able to trigger the innate immune system through binding to TLRs, which leads to the killing and clearance of viruses, even though it potentially capable of harming the host because of sustainable inflammatory conditions [15]. Studies have reported that signaling pathways by TLRs might be involved in the pathogenesis of SARS‐CoV‐2 as previous investigations have reported the involvement of TLRs in the pathogenesis of the Middle East respiratory syndrome (MERS) as well as SARS‐CoV [9]. It was also reported that COVID‐19 severity was associated with IL‐6 levels, which could be potentially linked to the stimulation of signaling by TLRs. SARS‐CoV‐2 stimulated TLRs that culminated in inflammasome activation and IL‐1β production, which is able to trigger IL‐6 production [16]. In addition, TLR signaling through Janus kinase (JAK)-Signal transducer and activator of transcription (STAT) might result in macrophage activation syndrome. As a consequence, TLRs play a dual role during infection by viruses [17–19].

In the current investigation, we determined the expression levels of TLR3, TLR7, TLR8, and TLR9 in the epithelial cells obtained from confirmed COVID-19 cases with and without different clinical symptoms.

## Materials and methods

### Study population

**Fig. 1:**
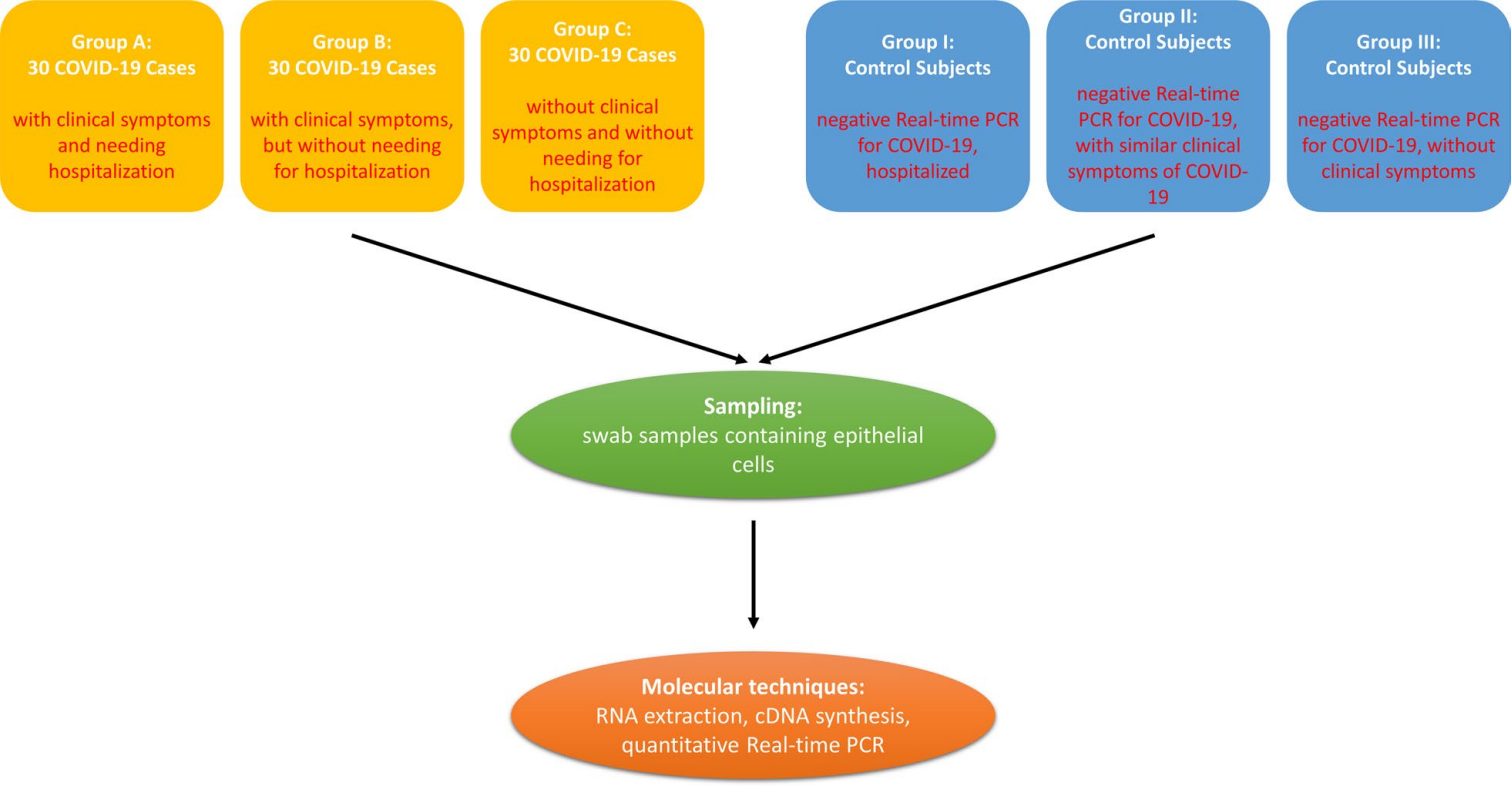
The flow-diagram of study subject selection and study procedure

The experiment population in the present investigation contained a total of 90 patients with COVID-19 referred to the Ali Ibn Abi Talib Hospital, Rafsanjan University of Medical Sciences, Rafsanjan, Iran during December 2020 to July 2021. Diagnosis of COVID-19 was conducted based on the detection of SARS-CoV-2 genetic content in the nasopharyngeal swab samples by Real-Time PCR test, computerized tomography (CT) scan of chest for detection of COVID-19 patterns, and typical clinical manifestations of the patients [20]. For sampling, the swab was inserted about 8–10 cm from the nostril to the posterior wall of the nasopharynx and then were rotated about 5–10 times and left in place for 5 s to collect the specimen [21]. Sample collection, CT scan, and clinical evaluations (like determination of Oxygen saturation) were implemented in the first hospital arrival of the subjects. The patients were categorized into three groups based on the severity of the clinical symptoms and needing for hospitalization: Group A; 30 subjects with COVID-19 infection (positive results of Real-time PCR test and confirmed CT scan) with clinical symptoms and needing hospitalization. Group B; 30 subjects with COVID-19 infection (positive result of Real-time PCR test and confirmed CT scan) with clinical symptoms, but without needing for hospitalization. Group C; 30 subjects with COVID-19 infection (positive result of Real-time PCR test and confirmed CT scan) without clinical symptoms. As the control group, 50 age- and sex-matched individuals were enrolled who were not confirmed for SARS-CoV-2 infection through Real-time PCR and CT scan results. The control groups were categorized into three groups: I; Subjects without COVID-19, but hospitalized due to similar clinical symptoms as COVID-19 (such as coughing, fever, pain, etc.). II; Subjects without COVID-19 and with similar clinical symptoms as COVID-19 (such as coughing, fever, pain, etc.), but without necessity for hospitalization. III; Subjects without COVID-19, without any clinical symptoms, and without necessity for hospitalization. Controls were checked by Real-time PCR, CT scan, as well as clinical manifestations to identify and confirm infection with SARS-CoV-2. None of the control subjects had immune-related disorders, such as autoimmune diseases, allergy and cancer, or liver diseases. Control Groups I and II had similar clinical symptoms like COVID-19 probably due to adult cold or sessional Influenza infection, but confirmed as negative for COVID-19 through Real-time PCR and CT scan results. The demographic data, laboratory indices, and clinical presentations of the study participants are listed in Table 2. The flow-diagram of study subject selection and study procedure are shown in the Fig. 1 [22]. After evaluation of the patients for COVID-19 infection by molecular, CT scan, and clinical evaluations, samples were stored in − 80 °C until further evaluations (mRNA extraction, cDNA synthesis, and Real-time PCR). The ethics committee of Rafsanjan University of Medical Sciences approved the protocol of this study (IR.RUMS.REC.1401.013) and all subjects signed a written informed consent to participate in this study.

### RNA extraction, cDNA synthesis, and quantitative real-time PCR

RNA extraction from the swab samples containing epithelial cells was conducted using Trizol total RNA extraction kit (GeneAll, Korea) according to manufactures’ instructions. Determination of the relative absorbance ratio at A260/A280 and A260/A230 by a spectrophotometer (Nano Drop 2000, Thermo Scientific, USA) was exerted to assess the extracted RNA concentration and purity. Then, template RNA was reverse-transcribed by PrimeScript first strand cDNA Synthesis Kit (TAKARA, Japan) following the manufacturer’s guidelines using Thermal Cycler instrument (Eppendorf, Germany). Real-time mRNA expression of the target genes was conducted by SYBR green master mix (I&L Biosystems, England) using ABI StepOnePlus real-time PCR System (Applied Biosystems, Foster City, CA, USA) via specific primer sets (Table 1). Primers were designed using Primer Express 3.0.1 Software (ThermoFisher Scientific, USA). All primers were checked for accuracy and specificity by the Basic Local Alignment Search Tool (BLAST; http://www.ncbi.nlm.nih.gov/tools/primer-blast/). Production of primers was done using the custom oligonucleotide synthesis service Metabion (Martinsried, Germany). The Real-time analyses were conducted in triplicate order. The transcript level of β-Actin was measured as housekeeping gene to normalize the expression levels of target genes. The comparative threshold cycle method (2^−∆∆ct^) was exerted to measure the relative amounts of target genes in each sample [23].

**Table 1:** Primer sets used to determine the mRNA expression levels of TLRs by Real-time PCR

Gene	Forward primer (5′ → 3′)	Reverse primer (5′ → 3′)
TLR1	GAAGATTTCTTGCCACCCTAC	GAACACAATGTGCAGACTCTC
TLR2	CTGGACAATGCCACATAC	CTAATGTAGGTGATCCTG
TLR4	CAGAACTGCAGGTGCTGG	GTTCTCTAGAGATGCTAG
TLR6	CTATTGTTAAAAGCTTCCATTTTGT	ACCTGAAGCTCAGCGATGTAGTTC
Β-Actin	ACTTAGTTGCGTTACACCCTT	GTCACCTTCACCGTTCCA

### Statistical analysis

For statistical analysis of the data, the SPSS software for windows v. 23 (SPSS Inc., Chicago, IL, USA) was used to analysis of data. The Kolmogorov–Smirnov test was exerted to normality evaluation of Scale variables. Group comparisons of non-parametric variables were conducted via the Mann–Whitney *U* test. To determine the relationship between scale variables, Spearman’s correlations were used. For plotting the graphs, the GraphPad Prism version 8.00 for Windows (GraphPad Software, La Jolla, CA, USA) was applied. The study results were presented as mean ± Standard deviation (SD). *P* values < 0.05 were considered as statistically significant.

## Results

### Demographics of the study subjects

The baseline data and clinical and laboratory characteristics of the study population participated in the study are summarized in the Table 2. The COVID-19 group contained 90 patients, with a mean age of 49.2 ± 13.4 years old, involving 48 (53.3%) males and 42 (46.7%) females. In the control group, a total of 50 subjects containing 28 (56%) males and 22 (44%) females with a mean age of 48.3 ± 12.6 years old were included. The patient and control groups were age- and sex-matched.

**Table 2:** Demographics and clinical presentations of COVID-19 patients and control group

Trait	COVID-19 subjects (N = 90)	Controls (N = 50)
Gender; Male/ Female (N, %)	48 (53.3%)/ 42 (46.7%)	28 (56%)/ 22 (44%)
Smoker/ Non-smoker	41 (45.5%)/ 49 (54.5%)	21 (42%)/ 29 (58%)
Age (Year, mean ± SD)	49.2 ± 13.4	48.3 ± 12.6
Duration of COVID-19 (Day)	10.9 ± 1.4	–
Oxygen saturation	91.8 ± 7.1	–
Systolic BP (mmHg)	138.7 ± 20.4	–
Diastolic BP (mmHg)	75.8 ± 9.9	–
WBC (cells/mm^3^)	9451 ± 1877.3	–
Neutrophil–lymphocyte ratio	10.4 ± 6.9	–
ALP (IU/L)	244.8 ± 42.9	–
AST (IU/L)	35.7 ± 11.3	–
ALT (IU/L)	36.4 ± 7.5	–
LDH (IU/L)	408.2 ± 97.1	–
CRP (mg/L)	5.1 ± 1.2	–
ESR (mm/h)	22.7 ± 10.3	–
BMI (kg/m^2^)	27.9 ± 7.1	–
Total cholesterol (mg/dl)	223.2 ± 41.9	–
TG (mg/dl)	173.7 ± 51.5	–
LDL (mg/dl)	144.2 ± 35.6	–
HDL (mg/dl)	49.2 ± 14.1	–
Creatinine (mg/dl)	1.87 ± 0.49	–
BUN (mg/dl)	24.4 ± 13.2	–
FBS (mg/dl)	101.5 ± 32.7	–
D-dimer (ng/ml)	1.74 ± 0.21	–
Cardiovascular diseases	12 (13.3%)	–
Diabetes	11 (12.2%)	–
Hypertension	16 (17.8%)	–
Fever	60 (66.6%)	–
Cough	59 (65.5%)	–
Dyspnea	55 (61.1%)	–
Sputum	39 (43.3%)	–
Vomiting/diarrhea	33 (36.7%)	–
Methylprednisolone use	29 (32.2%)	–
Remdesivir use	21 (23.3%)	–
Azithromycin use	12 (13.3%)	–
Anticoagulation therapy	16 (17.8%)	–

*COVID-19* Coronavirus disease 2019, *WBC* white blood cell, *CRP* C-reactive protein, *ALP* alkaline phosphatase, *AST* aspartate aminotransferase, *ALT* alanine aminotransferase, *LDH* lactate dehydrogenase, *ESR* erythrocyte sedimentation rate, *BMI* body mass index, *FBS* fasting blood sugar, *TG* triglyceride, *LDL* low density lipoprotein, *HDL* high density lipoprotein, *BUN* blood urea nitrogen, *OR* odds ratio, *CI* confidence interval, *SD* standard deviation, *BP* blood pressure

### mRNA expression of TLRs

**Fig. 2:**
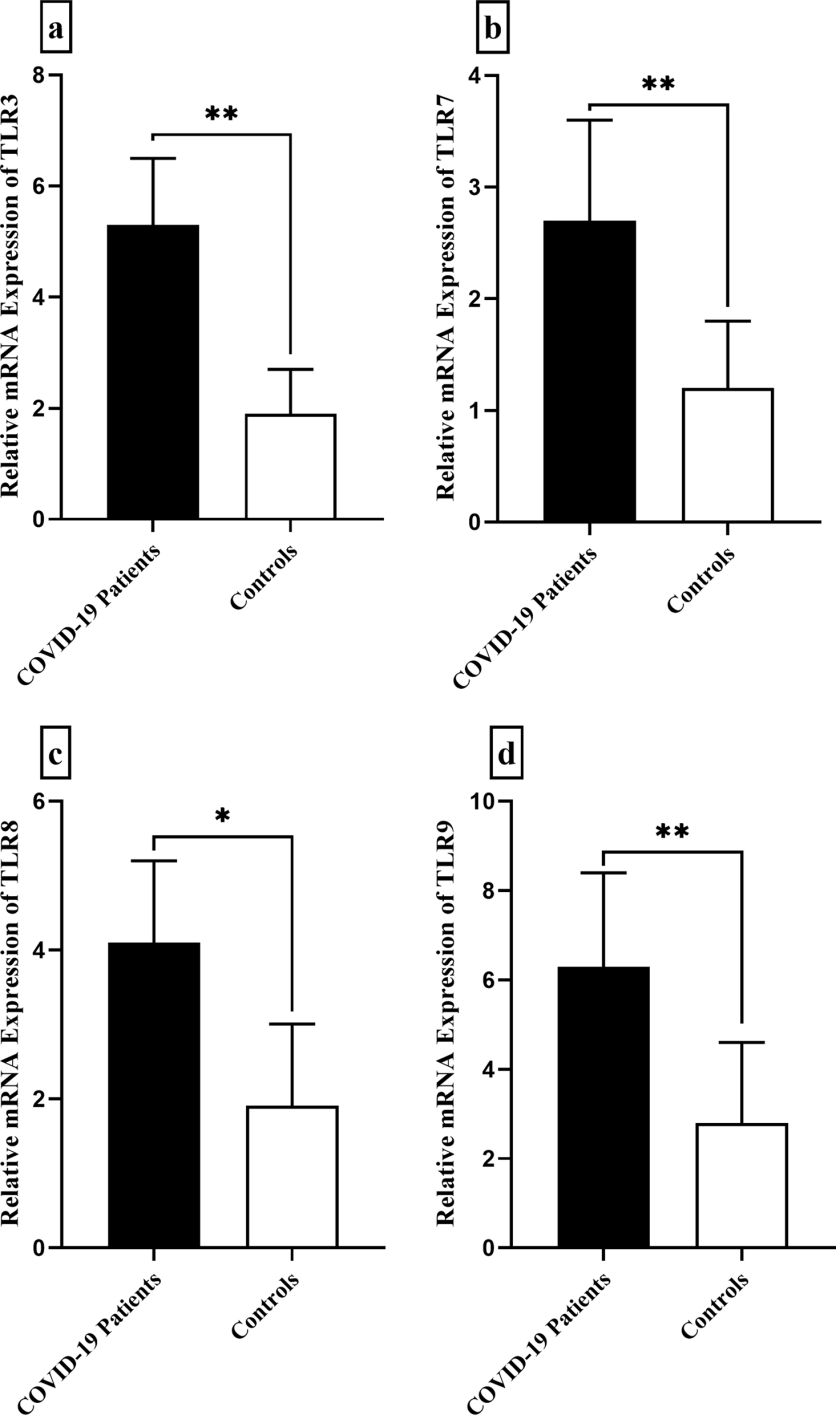
Bar graphs show the mRNA expression levels of TLR3 (**a**), TLR7 (**b**), TLR8 (**c**), and TLR9 (**d**) in the epithelial cells obtained from COVID-19 cases compared to the control group (* show *P* < 0.05 and ** shows a *P* < 0.01)

The overall analysis of the TLR expression indicated significant upregulation of TLR3 (fold change = 2.78, *P* = 0.007), TLR7 (fold change = 2.25, *P* = 0.003), TLR8 (fold change = 2.14, *P* = 0.028), and TLR9 (fold change = 2.25, *P* = 0.009) in the epithelial cells from COVID-19 patients compared to the control subjects (Fig. 2).

We evaluated the mRNA expression of TLRs in the epithelial cells isolated from each group of patients and controls as described in the method section. The mRNA expression of TLR3 (fold change = 3.4, *P* = 0.032), TLR7 (fold change = 2.0, *P* = 0.041), TLR8 (fold change = 2.8, *P* = 0.019), and TLR9 (fold change = 2.1, *P* = 0.016) was significantly upregulated in COVID-19- Group A compared with the Control-Group I (Table 3). There was a significant upregulation of mRNA of TLR3 (fold change = 2.9, *P* = 0.011), TLR7 (fold change = 2.2, *P* = 0.020), TLR8 (fold change = 2.9, *P* = 0.040), and TLR9 (fold change = 2.5, *P* = 0.001) in COVID-19-Group A in comparison to the Control-Group II (Table 3). It was seen that mRNA expression of TLR3 (fold change = 2.2, *P* = 0.004), TLR7 (fold change = 2.5, *P* = 0.038), TLR8 (fold change = 3.1, *P* = 0.018), and TLR9 (fold change = 3.0, *P* = 0.026) was significantly upregulated in the COVID-19- Group A in comparison to Control-Group III (Table 3). There was a significant upregulation of mRNA of TLR3 (fold change = 2.1, *P* = 0.022), TLR7 (fold change = 1.9, *P* = 0.025), TLR8 (fold change = 2.0, *P* = 0.039), and TLR9 (fold change = 2.9, *P* = 0.024) in COVID-19-Group B compared with the Control-Group I (Table 3). It was observed that mRNA expression of TLR3 (fold change = 2.3, *P* = 0.016), TLR7 (fold change = 2.1, *P* = 0.033), TLR8 (fold change = 2.3, *P* = 0.022), and TLR9 (fold change = 2.4, *P* = 0.019) was significantly upregulated in the COVID-19- Group B versus Control-Group II (Table 3). There was a significant upregulation of mRNA of TLR3 (fold change = 2.4, *P* = 0.017), TLR7 (fold change = 2.0, *P* = 0.009), TLR8 (fold change = 2.2, *P* = 0.004), and TLR9 (fold change = 2.9, *P* = 0.010) in COVID-19-Group B compared with the Control-Group III (Table 3).

**Table 3:** Relative mRNA expression of TLRs in different groups in this study

Item	TLR3 Fold change (*P* value)	TLR7 Fold change (*P* value)	TLR8 Fold change (*P* value)	TLR9 Fold change (*P* value)
COVID-19- Group A vs. Control-Group I	3.4 (0.032)	2.0 (0.041)	2.8 (0.019)	2.1 (0.016)
COVID-19- Group A vs. Control-Group II	2.9 (0.011)	2.2 (0.020)	2.9 (0.040)	2.5 (0.001)
COVID-19- Group A vs. Control-Group III	2.2 (0.004)	2.5 (0.038)	3.1 (0.018)	3.0 (0.026)
COVID-19- Group B vs. Control-Group I	2.1 (0.022)	1.9 (0.025)	2.0 (0.039)	2.2 (0.024)
COVID-19- Group B vs. Control-Group II	2.3 (0.016)	2.1 (0.033)	2.3 (0.022)	2.4 (0.019)
COVID-19- Group B vs. Control-Group III	2.4 (0.017)	2.0 (0.009)	2.2 (0.004)	2.9 (0.010)
COVID-19- Group C vs. Control-Group I	1.2 (0.099)	1.4 (0.18)	1.1 (0.087)	1.0 (0.891)
COVID-19- Group C vs. Control-Group II	1.2 (0.084)	1.2 (0.082)	1.0 (0.530)	1.1 (0.787)
COVID-19- Group C vs. Control-Group III	1.3 (0.20)	1.0 (0.139)	1.2 (0.449)	1.4 (0.094)

*TLR* toll-like receptor, *COVID-19* Coronavirus diseases 2019, *CI* Confidence interval

COVID-19-Group A; 30 subjects with COVID-19 infection with clinical symptoms and needing hospitalization. COVID-19-Group B; 30 subjects with COVID-19 infection with clinical symptoms, but without needing for hospitalization. COVID-19-Group C; 30 subjects with COVID-19 infection without clinical symptoms

Control-Group I; Subjects without COVID-19, but hospitalized due to similar clinical symptoms as COVID-19 (such as coughing, fever, pain, etc.). Control-Group II; Subjects without COVID-19 and with similar clinical symptoms as COVID-19 (such as coughing, fever, pain, etc.), but without necessity for hospitalization. Control-Group III; Subjects without COVID-19, without any clinical symptoms, and without necessity for hospitalization

There was no significant upregulation of TLR3, TLR7, TLR8, and TLR9 in the epithelial cells from COVID-19-Group C compared to Control-Group I, Control-Group II, and Control-Group III (Table 3).

The mRNA expression of TLRs was compared between different COVID-19 groups. It was seen that mRNA expression of TLR3 (fold change = 1.9, *P* = 0.027), TLR7 (fold change = 1.7, *P* = 0.047), TLR8 (fold change = 1.89, *P* = 0.029), and TLR9 (fold change = 1.7, *P* = 0.020) was significantly upregulated in the COVID-19-Group A versus COVID-19-Group C. However, transcript levels of TLRs did not have significant difference between COVID-19-Group A versus COVID-19-Group B as well as COVID-19-Group B versus COVID-19-Group C (data not shown).

### Correlation of TLRs expression with clinicopathological findings

The mRNA expression of TLRs in the epithelial cells from whole COVID-19 cases was evaluated in correlation with clinical and laboratory findings of the patients. There was a significant positive correlation between percentage of oxygen saturation and transcript levels of TLR3 (*rho* = 0.37, *P* = 0.041), TLR7 (*rho* = 0.30, *P* = 0.026), TLR8 (*rho* = 0.35, *P* = 0.022), and TLR9 (*rho* = 0.39, *P* = 0.028) in the overall COVID-19 cases. In addition, a significant positive correlation was detected between WBC count and transcript levels of TLR3 (*rho* = 0.55, *P* = 0.020), TLR7 (*rho* = 0.59, *P* = 0.029), TLR8 (*rho* = 0.44, *P* = 0.012), and TLR9 (*rho* = 0.41, *P* = 0.034) in the whole COVID-19 cases. There was positive significant correlation between Neutrophil–lymphocyte ratio and mRNA expression of TLR3 (*rho* = 0.62, *P* = 0.012), TLR7 (*rho* = 0.60, *P* = 0.013), TLR8 (*rho* = 0.49, *P* = 0.011), and TLR9 (*rho* = 0.39, *P* = 0.036) in all COVID-19 cases. There was a significant positive correlation between LDH level and mRNA expression of TLR3 (*rho* = 0.39, *P* = 0.041), TLR7 (*rho* = 0.41, *P* = 0.008), and TLR8 (*rho* = 0.49, *P* = 0.030) in the whole COVID-19 cases. A significant positive correlation was observed between CRP and mRNA expression of TLR3 (*rho* = 0.85, *P* = 0.001), TLR7 (*rho* = 0.80, *P* = 0.004), TLR8 (*rho* = 0.78, *P* = 0.010), and TLR9 (*rho* = 0.48, *P* = 0.035) in the all COVID-19 cases. There was positive significant correlation between ESR and mRNA expression of TLR3 (*rho* = 0.71, *P* = 0.005), TLR7 (*rho* = 0.62, *P* = 0.011), TLR8 (*rho* = 0.65, *P* = 0.029), and TLR9 (*rho* = 0.40, *P* = 0.044) in all COVID-19 cases (Table 4).

**Table 4:** Correlation analysis between the transcript level of TLRs and the baseline and clinical characteristics of the COVID-19 cases

Item	TLR3	TLR7	TLR8	TLR9
Age	*rho* = 0.23, *P* = 0.098	*rho* = 0.18, *P* = 0.254	*rho* = 0.16, *P* = 0.650	*rho* = 0.10, *P* = 0.309
Duration of COVID-19	*rho* = 0.29, *P* = 0.088	*rho* = 0.24, *P* = 0.074	*rho* = 0.25, *P* = 0.147	*rho* = 0.27, *P* = 0.205
Oxygen saturation	***rho* = 0.37, *P* = 0.041**	***rho* = 0.30, *P* = 0.026**	***rho* = 0.35, *P* = 0.022**	***rho* = 0.39, *P* = 0.028**
Systolic BP	*rho* = 0.11, *P* = 0.410	*rho* = 0.16, *P* = 0.201	*rho* = 0.20, *P* = 0.085	*rho* = 0.13, *P* = 0.416
Diastolic BP	*rho* = 0.11, *P* = 0.265	*rho* = 0.21, *P* = 0.247	*rho* = 0.13, *P* = 0.497	*rho* = 0.20, *P* = 0.122
WBC	***rho* = 0.55, *P* = 0.020**	***rho* = 0.59, *P* = 0.029**	***rho* = 0.44, *P* = 0.012**	***rho* = 0.41, *P* = 0.034**
Neutrophil–lymphocyte ratio	***rho* = 0.62, *P* = 0.012**	***rho* = 0.60, *P* = 0.013**	***rho* = 0.49, *P* = 0.011**	***rho* = 0.39, *P* = 0.036**
ALP	*rho* = 0.16, *P* = 0.211	*rho* = 0.10, *P* = 0.854	*rho* = 0.19, *P* = 0.099	*rho* = 0.18, *P* = 0.388
AST	*rho* = 0.18, *P* = 0.248	*rho* = 0.15, *P* = 0.411	*rho* = 0.10, *P* = 0.815	*rho* = 0.13, *P* = 0.867
ALT	*rho* = 0.12, *P* = 0.381	*rho* = 0.09, *P* = 0.880	*rho* = 0.19, *P* = 0.231	*rho* = 0.19, *P* = 0.299
LDH	***rho* = 0.39, *P* = 0.041**	***rho* = 0.41, *P* = 0.008**	***rho* = 0.49, *P* = 0.030**	*rho* = 0.25, *P* = 0.090
CRP	***rho* = 0.85, *P* = 0.001**	***rho* = 0.80, *P* = 0.004**	***rho* = 0.78, *P* = 0.010**	***rho* = 0.48, *P* = 0.035**
ESR	***rho* = 0.71, *P* = 0.005**	***rho* = 0.62, *P* = 0.011**	***rho* = 0.65, *P* = 0.029**	***rho* = 0.40, *P* = 0.044**
BMI	*rho* = 0.13, *P* = 0.655	*rho* = 0.11, *P* = 0.249	*rho* = 0.08, *P* = 0.709	*rho* = 0.16, *P* = 0.380
Total cholesterol	*rho* = 0.20, *P* = 0.568	*rho* = 0.09, *P* = 0.744	*rho* = 0.20, *P* = 0.516	*rho* = 0.16, *P* = 0.246
TG	*rho* = 0.13, *P* = 0.214	*rho* = 0.19, *P* = 0.501	*rho* = 0.25, *P* = 0.077	*rho* = 0.16, *P* = 0.504
LDL	*rho* = 0.09, *P* = 0.209	*rho* = 0.19, *P* = 0.640	*rho* = 0.15, *P* = 0.309	*rho* = 0.14, *P* = 0.506
HDL	*rho* = 0.11, *P* = 0.246	*rho* = 0.16, *P* = 0.407	*rho* = 0.15, *P* = 0.230	*rho* = 0.16, *P* = 0.488
Creatinine	*rho* = 0.26, *P* = 0.087	*rho* = 0.11, *P* = 0.501	*rho* = 0.13, *P* = 0.249	*rho* = 0.18, *P* = 0.202
BUN	*rho* = 0.11, *P* = 0.480	*rho* = 0.23, *P* = 0.509	*rho* = 0.11, *P* = 0.600	*rho* = 0.14, *P* = 0.407
FBS	*rho* = 0.13, *P* = 0.260	*rho* = 0.18, *P* = 0.354	*rho* = 0.08, *P* = 0.580	*rho* = 0.18, *P* = 0.404
D-dimer	*rho* = 0.08, *P* = 0.333	*rho* = 0.16, *P* = 0.240	*rho* = 0.11, *P* = 0.266	*rho* = 0.25, *P* = 0.088

Bold values show statistically significant comparisons

*TLR* toll-like receptor, *COVID-19* Coronavirus disease 2019, *WBC* white blood cell, *CRP* C-reactive protein, *ALP* alkaline phosphatase, *AST* aspartate aminotransferase, *ALT* alanine aminotransferase, *LDH* lactate dehydrogenase, *ESR* erythrocyte sedimentation rate, *BMI* body mass index, *FBS* fasting blood sugar, *TG* triglyceride, *LDL* low density lipoprotein, *HDL* high density lipoprotein, *BUN* blood urea nitrogen, *OR* odds ratio, *CI* confidence interval, *BP* blood pressure

We performed correlation analysis between each COVID-19 group with the clinical and laboratory findings of the patients. It was observed that oxygen saturation, WBC count, Neutrophil–lymphocyte ratio, LDH level, CRP level, and ESR level had a significant positive correlation with the transcript levels of TLR3, TLR7, TLR8, and TLR9 in the COVID-19 Group A as well as COVID-19 Group B patients. However, mRNA expression of TLRs did not correlate significantly with the clinical and laboratory findings of the COVID-19 Group C patients (data not shown).

## Discussion

Several studies have suggested the role of TLRs in enhancing humoral responses during COVID-19 infection, but so far, no study has examined the expression of TLR3, TLR7, TLR8 and TLR9 on respiratory epithelial cells from COVID-19 patients. Here in the current investigation, we attempted to measure the mRNA expression levels of TLR3, TLR7, TLR8, and TLR9 in the nasopharyngeal epithelial cells from COVID-19 patients in order to determine the role of these innate immune system molecules in the inflammatory settings of COVID-19 patients. Our experiments revealed that expression of TLRs were increased in the epithelial cells from COVID-19 subjects at different settings of clinical presentations and requirement for hospitalization in comparison to control group. In addition, increased expression of TLRs was associated with disease severity of the patients.

Since SARS-CoV2 proliferates within the cytoplasm of airway epithelial cells and subsequently infects cells in the lungs, innate immune system responses within airway epithelial cells play a crucial role in the COVID-19 infection. Hence, the innate immune system responses play a critical role in clearing the virus or continue to infect lung cells [9, 24]. Therefore, studying the expression of TLRs inside the epithelial cells of the respiratory tract can help us to identify appropriate immune responses against the SARS-CoV2.

TLR7 was shown to recognize the complex made by binding of the S protein of SARS-CoV2 to ACE2. A number of immune cells, such as macrophages, monocyte, and DCs express TLR7 and its activation results in generation of several inflammatory mediators, including monocyte chemoattractant protein‐1 (MCP-1), macrophage inflammatory protein-1 alpha (MIP-1α, also called CCL3), TNF‐α, type 1 IFN, IL‐1, IL‐6 [25]. Moreover, in 4 male subjects with severe forms of COVID‐19, loss of function variants of X‐chromosomal TLR7 were identified infection that resulted in a deficiency in the responses by type 1 and 2 IFNs [26]. It has been suggested that TLR7 is able to stimulate the NET formation in COVID‐19 subjects and triggering of the TLR7/8 complex is capable of inducing powerful pro‐inflammatory response in COVID-19 patients, culminating in acute lung injury. As a result, TLR7 activation might play controversial roles during COVID-19 progression [27, 28]. Inhibitors of mammalian target of rapamycin (mTOR) interfere with the signaling pathways associated with MyD88, IRF7, and TLR9. It was observed that blocking of mTOR and activation of p53 might confer therapeutic outcomes in patients with COVID‐19 [29]. It was also revealed that TLR4 genetic polymorphisms might be associated with a severe form of COVID-19 and in-hospital mortalities [30]. Therefore, it seems that alteration in the signaling of TLRs might determine the disease severity in the COVID-19 cases.

Our study indicated that the mRNA expression of TLRs was upregulated in the nasopharyngeal epithelial cells from COVID-19 patients compared to the controls. More specifically, TLR3, TLR7, TLR8, and TLR9 were upregulated in the COVID-19 cases with clinical symptoms and needing hospitalization as well as in those with clinical symptoms but without requirement for hospitalization for supportive cares. That notwithstanding, we did not observe any significant modification in the transcript levels of TLRs in the nasopharyngeal epithelial cells obtained from COVID-19 subjects without clinical symptoms in comparison to all control groups.

Studies show that COVID-19 patients with severe form of disease hospitalized in the ICU show hallmarks of hyperactivation of TLRs with increased levels of agonists of nucleic acid-sensing TLRs found in the blood and lungs samples. Using nanoparticle- or microfiber-based approaches for Damage associated molecular pattern (DAMP)/PAMP scavenging might be beneficial in confining SARS-CoV-2-stimulated hyperinflammation and improving the outcomes in the COVID-19 patients with higher disease severity [31].

Our investigation demonstrated that the expression of TLRs in the nasopharyngeal epithelial cells was not significantly different between COVID-19 subjects without clinical symptoms in comparison to all control groups. Hence, hyperactivation of TLRs might occur in the severe form of COVID-19 disease. Furthermore, detected that mRNA expression of TLR3, TLR7, TLR8, and TLR9 were correlated with the levels of inflammation (as demonstrated CRP and ESR levels) and potentially tissue damage (as represented by levels of and LDH). Moreover, levels of mRNA expression of TLRs were correlated with levels of oxygen saturation in the COVID-19 patients. As a consequence, it appears that aberrant TLR signaling plays a role in the exacerbation of the patient’s clinical presentations as well as in the requirement for supportive cares in hospital facilities. Hence, therapeutic approaches targeting the signaling of TLRs along with other options might be effective in controlling sever forms of COVID-19.

In this study, we selected subjects without SARS-CoV-2 infection who had some similar symptoms as COVID-19 patients with and without requirement for hospitalization. Comparing the COVID-19 subjects with these groups indicated that TLRs expression was upregulated in confirmed cases with SARS-CoV-2 infection. When comparing this evidence with the control group without symptoms and necessity for hospitalization, it appears that TLRs are probably upregulated in COVID-19 cases (with unique clinical presentations) and may result in severe forms of the disease that necessitate subjects to be hospitalized to receive supportive cares.

Even though we tried to perform the study in an utmost logical design, however there are a number of limitations and caveats that should be addressed. Bias may occur based on the selection of the target patients and how the specimens were collected. Therefore, we decided to include maximum possible of sample size to decrease such biases. Moreover, we tried to abrogate potential biases through evaluation of COVID-19 cases by molecular, imaging, and clinical presentations of the subjects. Moreover, we evaluated the transcript level of TLRs in a pool of nasopharyngeal cells and did not discriminate it for different epithelial cells like club and ciliated cells that may possess diverse expression levels of TLR mRNAs. We also recommend blotting approaches in the future studies for identification of protein expression of TLRs on the epithelial cells obtained from COVID-19 patients.

In conclusion, our investigation indicated that the mRNA expressions of TLR3, TLR7, TLR8, and TLR9 are upregulated in the nasopharyngeal epithelial cells from COVID-19 patients. Upregulation of TLRs might be involved in the severity of the clinical symptoms. Moreover, aberrant expression of TLRs in the epithelial cells might show the need for supportive cares in the COVID-19 subjects. At the moment, it is not clear if hyperactivation of TLRs is a result of intensive disease (probably through other molecules and receptors of the immune system) or upregulation of TLRs results in severe forms of disease. Nonetheless, severe forms of COVID-19 associates with poor outcomes or hospitalization in ICU or even in-hospital mortality. In line with obtaining comprehensive data regarding the involvement of TLRs in the COVID-19, assessment of the mRNA levels of TLRs in the nasopharyngeal swab samples might contribute to the earlier detection of severe disease form and help to initiate the interventions to promote the patient outcomes. Additionally, devising therapeutics to antagonize TLRs as a treatment approach in the COVID-19 patients might hopefully open up new path in surviving those with sever forms of the disease.

## Data Availability

All data that support the conclusions of this manuscript are included within the article.
